# Analysis of gait characteristics and related factors in patients with Parkinson's disease based on wearable devices

**DOI:** 10.1002/brb3.3440

**Published:** 2024-03-27

**Authors:** Hongyin Tang, Xianglian Liao, Jian Yao, Yilan Xing, Xin Zhao, Weibin Cheng, Tianxiang Gu, Yan Huang, Guang Xu, Ping Luan, Junzhang Tian, Guihua Li

**Affiliations:** ^1^ Department of Neurology Guangdong Second Provincial General hospital Guangzhou Guangdong China; ^2^ The Second School of Clinical Medicine Southern Medical University Guangzhou Guangdong China; ^3^ Institute for Healthcare Artificial Intelligence Application Guangdong Second Provincial General Hospital Guangzhou Guangdong China; ^4^ Guangdong Second Provincial General hospital, Alzheimer's Disease Clinical Research Center Guangzhou Guangdong China; ^5^ School of Basic Medical Sciences Shenzhen University Shenzhen Guangdong China

**Keywords:** Parkinson's disease, postural instability and gait disorder dominant (PIGD), related factors

## Abstract

**Background:**

Postural instability and gait disorder dominant (PIGD) is one of the most common disabling symptoms of Parkinson's disease (PD), which seriously affects patients’ quality of life. Therefore, it is essential to identify PIGD and develop targeted interventions to reduce the risk of PIGD in PD patients.

**Aim:**

This study aimed to investigate the gait characteristics of PD patients based on wearable devices and to establish a predictive model for their related influencing factors.

**Methods:**

The retrospective medical records of patients from January 2020 to September 2023 were collected, including 159 patients with PD (divided into PIGD [*n* = 73] and non‐PIGD [*n* = 86] groups) and 200 healthy patients (as the healthy control group). Information from social demographic data, a blood test, scale scores, gait analysis based on wearable devices, white matter lesions, and the Fazekas scale was extracted and analyzed.

**Results:**

Compared with the healthy control group, the mean step length, mean rate, mean angular velocity, and step length were lower in the PD group, while the mean steps were higher in the turning test. The incidence of PIGD was 46% in PD patients, and PD patients with the non‐tremor onset mode were more likely to develop PIGD than those with the tremor onset mode. Compared to the non‐PIGD group, the PIGD group showed more serious gait problems in different experimental tasks and had a higher Hoehn and Yahr (H‐Y) stage, Hamilton Anxiety Scale (HAMA) score, Hamilton Depression Scale score, periventricular white matter (PVWM) score, deep white matter score, and Fazekas scale score, but they had lower hemoglobin levels, D‐dimer levels, Tinetti Balance scores, Tinetti Gait scores, Berg Balance Scale scores, and Mini‐Mental State Examination (MMSE) scores. Logistic regression analysis showed that the MMSE score was negatively correlated with the occurrence of PIGD, while the HAMA score, H‐Y stage, PVWM score, and non‐tremor form of onset were positively correlated with the occurrence of PIGD

**Conclusion:**

The incidence of gait disorder in PD patients is higher than that in the normal population. Moreover, cognitive dysfunction, anxiety state, H‐Y stage, PVWM score, and the non‐tremor mode of onset can be considered independent risk factors for PIGD.

## Graphical abstract

To investigate the postural characteristics of PD patients and to establish a predictive model for their related influencing factors according to the information from social demographic data, a blood test, scale scores, gait analysis, white matter lesions, and the Fazekas scale.

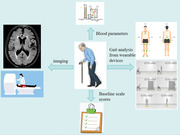

## 1 INTRODUCTION

Postural instability and gait disorder dominant (PIGD) is one of the most common disabling symptoms of Parkinson's disease (PD). In its early stage, it is mainly characterized by reduced swing amplitude on the affected side or in both arms and increased gait variability, such as changes in step length and walking time (Wu et al., 2015). With the progression of the disease to the middle and late stages, increasing gait variability, gait freezing, panic gait, and other manifestations can occur. Gait disorders in the middle and late stages increase the risk of falls, fractures, and even death (Mirelman et al., 2019). Therefore, it is essential to identify the occurrence of gait disorders to allow for timely interventions.

Scale assessment is often used in the clinic to assess gait disorders in PD patients; however, it is limited by its lack of sensitivity to changes in disease progression and its difficulty in capturing subtle movements. Studies (Naghavi et al., 2019; Tripoliti et al., 2013) have confirmed that wearable devices have high specificity and sensitivity for the early diagnosis and differential diagnosis of PD and that they can quantify a variety of gait characteristics (such as speed, variability, and asymmetry) to improve the effectiveness of clinical diagnosis. As people pay an increasing amount of attention to health and as science and technology continue to mature, intelligent devices have become more widely used in disease monitoring.

Because the postural gait pattern of PD is complex and influenced by many factors, many studies (Gallardo et al., 2014; Monaghan et al., 2023) around the world have investigated freezing of gait (FOG)‐related risk factors in PD patients. They have shown that Hoehn and Yahr (H‐Y) stage, anxiety and depression, cognitive dysfunction, and white matter vascular damage can affect the gait performance of PD patients, but the research conclusions are inconsistent. It is essential to identify high‐risk PD populations and develop targeted interventions that can reduce the incidence of FOG and fall events caused by gait disorders. Therefore, to better understand the gait characteristics of PD patients and their influencing factors, we conducted a clinical evaluation survey and assessment based on the wearable devices of PD patients who were hospitalized at the neurology clinic of the Second People's Hospital of Guangdong Province from January 2020 to September 2023. Next, we established a preliminary prediction model for the PD postural gait to provide a reference for the early detection, diagnosis, and treatment of gait disorders in PD patients and to reduce the risk of adverse events, such as FOG and falls.

## 2 MATERIALS AND METHODS

### 2.1 Subjects

A retrospective observational research study was conducted in the departments of the Second People's Hospital of Guangdong Province from January 2020 to September 2023, with 159 PD patients who met the criteria for idiopathic PD diagnosed according to the clinical criteria of the Movement Disorder Society Unified‐Parkinson Disease Rating Scale (MDS‐UPDRS) and were confirmed by two senior physicians. For the same period, 200 age‐matched patients without PD were selected as the control group. The inpatients came from Guangdong province, Hong Kong, and other districts and areas in southern China. The inclusion criteria were as follows: patients who met the diagnostic criteria for primary PD and patients who completed all survey scales and provided basic clinical data. The exclusion criteria were as follows: other types of parkinsonism, such as secondary parkinsonism, parkinsonism superimposed syndrome, and familial parkinsonism; other diseases with gait disturbance (e.g., spinal joint injury, muscle spasm, stroke, peripheral neuropathy, muscular diseases, hydrocephalus, and cognitive impairment); organ (e.g., heart, lung, liver, and kidney) failure, malignant tumor, unstable condition, or serious internal diseases; severe behaviors or psychosis; or uncooperative behavior.

This study was approved by the ethics committee of the Second People's Hospital of Guangdong Province. There was a minimal risk of loss of confidentiality for this study.

### 2.2 Contents and methods

All participants provided their written informed consent and completed an epidemiological data survey that included demographics and epidemiological information, gait assessment scales, hematological assessments, wearable device evaluation, and imaging evaluation, as discussed below.

#### 2.2.1 Demographics and epidemiology

Name, gender (male/female), age (years), age at onset (years), disease duration (years), mode of onset (tremor type/non‐tremor type), marital status, educational level, use of anti‐Parkinson's drugs, and other pieces of general information were obtained via the survey.

#### 2.2.2 Gait assessment scales

These included the MDS‐UPDRS (I, II, III) components, H‐Y stage, Mini‐Mental State Examination (MMSE), Pittsburgh Sleep Quality Index, Hamilton Anxiety Scale (HAMA), Hamilton Depression Scale (HAMD), non‐motor symptoms scale (NMSS), Parkinson's Disease Sleep Scale, Tinetti Balance, Tinetti Gait, Berg Balance Scale (BBS), and Patient Assessment of Constipation Symptoms (PAC‐SYM).

#### 2.2.3 Hematological assessments

Data related to C‐reactive protein, D‐Dimer, free triiodothyronine, free thyroxine, thyroid‐stimulating hormone, uric acid (UA), glycosylated hemoglobin, electrolytes (Fe, Cl, Ca, P, Mg, and K), homocysteine (HCY), white blood cells (WBC), red blood cells (RBC), hemoglobin (HGB), and platelets (PLT) were obtained.

#### 2.2.4 Wearable devices

A GYENNO MATRIX wearable device (GYENNO SCIENCE, Shenzhen, China, MA200) was used to conduct the timed up and go (TUG) test, narrow lane test, and turning test on the enrolled patients. During the TUG test, the patients were instructed to stand up from a chair, walk in a straight line for 5 m at a comfortable speed, turn 180° around at the 5‐m marker, walk back to the starting point, turn 180° around in front of the chair, and sit down on the chair. During the narrow lane test, the patients were instructed to go through a narrow passage that was two fists wider than they were and walk in a straight line for 5 m at a comfortable speed. During the turning test, the patients were instructed to turn in a circle twice to the left and twice to the right. The patients were tested at their usual normal pace in all of the tests. We monitored and recorded the relevant objective data, including the standing‐up time, walking time, sitting time, step length, left and right foot speed, freezing time, and turning time. The patients were tested during OFF medication, because there were more serious gait disorders.

#### 2.2.5 Imaging evaluation

All PD patients underwent improved head magnetic scanning (including TI, T2, FLAIR, DWI), and the extent and severity of white matter vascular lesions were evaluated using the modified Fazekas method based on MRI‐T2WI or FLAIR sequence images. Fazekas scale scores were calculated on a scale of 0−6: A (periventricular white matter, PVWM) +B (deep white matter, DWM) algorithm was used. Fazekas grading: A+B (level 1, 2, 3) for judgment.

### 2.3 Statistical analysis

In this study, SPSS Statistics 23.0 was used for statistical analyses of the data, and basic descriptive statistical analysis was conducted using the frequency method. Continuous variables were expressed as the mean ± SD or median (25−75 percentiles), whereas categorical variables were expressed as percentages (%). A Student's *t*‐test was used for normally distributed continuous variables, whereas the Mann–Whitney U test was applied to continuous variables without a normal distribution or level variable. The χ2 test was used for categorical variables. Univariate logistic regression was used for univariate analysis, least absolute shrinkage and selection operator (LASSO) regression was used for variable screening, and the selected variables were included in multivariate logistic regression to establish a prediction model for PIGD. The predictive effect of the model was evaluated using a receiver operating characteristic (ROC) curve and the area under the curve (AUC) value, and *p* ≤.05 was considered statistically significant.

## 3 RESULTS

We recruited a total of 159 patients (93 males and 66 females) with PD as the case group, with an average age of 66.64 ± 11.09 years, and we matched 200 normal patients (98 males and 102 females) as the control group, with an average age of 61.21 ± 11.64 years. Based on the mean tremor component scores (2.10, 3.15a, 3.15b, 3.16a, 3.16b, 3.17a, 3.17b, 3.17c, 3.17d, 3.17e, and 3.18) of the MDS‐UPDRS divided by the mean postural gait component scores (2.12, 2.13, 3.10, 3.11, and 3.12), all of the PD patients were grouped into either the PIGD group (mean value ≤ 0.90) or the non‐PIGD group (mean value > 0.90). There were 73 patients in the PIGD group, with an average age of 66.70 ± 10.92 years, and 86 patients in the non‐PIGD group, with an average age of 63.90 ± 10.71 years.

We analyzed the data of the PD and healthy control groups by comparing their basic demographics and scale scores (Table 1), and we found that there were no significant differences in age (y), weight (kg), height (cm), sex ratio (male/female), smoking status, or alcohol consumption between the PD and control groups (all *p* > .05). Compared with the control group, the PD group had lower MMSE, Tinetti Balance, Tinetti Gait, and BBS scores, but higher HAMA, HAMD, and PAC‐SYM scores (all *p *< .05). We also compared the gait parameters of these two groups (Table 1) using a commercially available wearable motion and gait quantification assessment system known as the MATRIX. The kinematic and dynamic parameters of human movement were collected by sensor devices placed at 10 data nodes: the chest, waist, left and right wrists, left and right thighs, left and right lower legs, and left and right feet. These parameters were transmitted to the operation center in real time using wireless transmission technology for three‐dimensional movement posture reconstruction. Based on these data, the gait, posture balance, arm swing, and whole‐body movement coordination were assessed. We found that compared with the control group, in the PD group, the mean step length, mean rate, and step length were lower in the timed up‐and‐go and narrow track tests (*p *< .05), while the mean steps was higher in the turning test (*p *< .05). Furthermore, the mean angular velocity was lower in the PD group than in the control group (*p *< .05).

**TABLE 1:** Comparison of the basic demographics, scale scores, and gait parameters in the Parkinson's disease (PD) and healthy control groups.

		Characteristic	Control	PD	*t*	*p*
Baseline clinical characteristics		Sex (male/female)	98/102	93/66	2.060	.151
		Height (cm, $\bar{x}$ ± s)	158.79 ± 7.84	159.79 ± 7.18	0.124	.472
		Age (years, $\bar{x}$ ± s)	61.21 ± 11.64	66.64 ± 11.09	0.124	.052
		Weight (kg, $\bar{x}$ ± s)	59.75 ± 9.28	60.23 ± 6.83	0.471	.419
		Smoking history	124/76	89/70	0.054	.121
		Alcohol consumption	28/172	29/130	0.296	.458
Baseline scale scores		MMSE	25.11 ± 2.83	21.74 ± 4.91	−5.067	< .001^*^
		HAMA	6.42 ± 4.80	9.92 ± 3.92	4.647	< .001^*^
		HAMD	7.39 ± 3.85	9.44 ± 4.74	2.640	.009^*^
		PSQI	8.58 ± 4.70	8.52 ± 3.95	−0.089	.929
		Tinetti gait	11.30 ± 0.72	9.93 ± 1.68	−6.560	< .001^*^
		Tinetti balance	15.34 ± 0.68	14.14 ± 1.38	−6.720	< .001^*^
		BBS	54.00 ± 1.21	46.87 ± 6.56	−9.651	< .001^*^
		PAC‐SYM	9.85 ± 5.69	12.25 ± 7.04	2.188	.031^*^
Gait analysis from wearable devices	Timed up and go	Mean step length	45.06 ± 7.72	40.44 ± 10.73	−2.253	.024^*^
		Mean stride velocity	0.80 ± 0.17	0.69 ± 0.23	−2.964	.003^*^
		Stride length	89.18 ± 15.05	80.16 ± 21.22	−2.300	.021^*^
		Mean step frequency	106.11 ± 10.71	102.30 ± 12.70	−1.435	.151
		Mean stride time	1.15 ± 0.12	1.21 ± 0.16	−1.239	.215
		Double support	0.21 ± 0.04	0.23 ± 0.07	−1.626	.104
	Narrow‐track test	Mean step length	44.47 ± 8.10	37.41 ± 11.25	−3.275	.001^*^
		Mean stride velocity	0.81 ± 0.19	0.65 ± 0.23	−3.739	< .001^*^
		Stride length	88.29 ± 16.18	74.57 ± 22.49	−3.093	.002^*^
		Mean step frequency	108.49 ± 11.08	104.08 ± 12.48	−2.133	.033^*^
		Mean stride time	1.13 ± 0.12	1.19 ± 0.15	−1.824	.068
		Double support	0.20 ± 0.04	0.23 ± 0.07	−2.603	.009^*^
	Turning test	Turning left duration	10.35 ± 3.90	23.47 ± 27.33	−5.716	< .001^*^
		Turning right duration	10.15 ± 3.76	23.73 ± 34.93	−5.013	< .001^*^
		Mean duration	10.25 ± 3.67	23.84 ± 30.49	−5.713	< .001^*^
		Mean steps	14.20 ± 3.78	30.71 ± 24.05	−5.661	< .001^*^
		Mean angular velocity	78.14 ± 22.96	47.17 ± 22.82	−6.521	< .001^*^

Abbreviations: BBS, Berg Balance Scale; MMSE, Mini‐Mental State Examination; HAMA, Hamilton Anxiety Scale; HAMD, Hamilton Depression Scale; PSQI, Pittsburgh Sleep Quality Index; PAC‐SYM, Patient Assessment of Constipation Symptoms.

* *p *< .05; *p*‐value is the result of *t*‐test and χ2 test.

We further compared the PIGD and non‐PIGD groups in terms of their basic data, blood parameters, and scale scores (Table 2). Of the 159 PD patients, 46% (73) had PIGD. PD patients with a non‐tremor onset mode were more likely to develop PIGD than those with a tremor onset mode. Compared with the non‐PIGD group, the PIGD group had a higher H‐Y stage, NMSS score, HAMA score, and HAMD score (*p *<.05), as well as a lower HGB level, D‐dimer level, Tinetti Balance score, Tinetti Gait score, BBS score, and MMSE score (*p *< .05). We also compared the gait parameters and evaluations of the white matter lesions of these two groups (Table 3), and we found that compared with the non‐PIGD group, PIGD patients had a lower mean step frequency and a longer mean stride time in the narrow‐track test. In the turning test, PIGD patients had a longer mean duration and mean steps and a smaller mean angular velocity. We also found that, compared with the non‐PIGD group, patients in the PIGD group had higher PVWM, DWM, and Fazekas scale scores based on MRI FLAIR images.

**TABLE 2:** Comparison between the postural instability and gait disorder dominant (PIGD) and non‐PIGD groups in terms of their basic data, blood parameters, scale scores, and gait analysis indexes obtained from wearable devices.

		Characteristic	PIGD	Non‐PIGD	*T*	*p*
Baseline clinical characteristics		Sex (male/female)	42/31	51/35	0.100	.752
		Age (years)	66.70 ± 10.92	63.90 ± 10.71	0.819	.418
		Duration of disease (years)	3.96 ± 2.97	4.49 ± 4.75	0.824	.411
		H‐Y stage	3.16 ± 0.98	2.24 ± 0.98	−5.903	< .001^*^
		Smoking history(yes/no) Alcohol consumption(yes/no) Hypertension(yes/no)	20/53 8/65 42/31	22/64 18/68 50/36	0.208 0.385 −0.623	.834 .698 .531
		Diabetes (yes/no) Coronary disease(yes/no) Hypercholesterolemia(yes/no) Cerebral hemorrhage(yes/no)	20/53 16/57 18/55 4/69	23/63 14/72 27/59 5/81	−0.771 −0.884 0.660 0.165	.439 .375 .507 .868
		Cerebral infarction(yes/no)	58/15	60/26	−0.391	.694
		Mode of onset (n/%) Tremor Non‐tremor	14 (19.18%) 59 (80.82%)	55 (63.95%) 31 (36.05%)	25.144	< .001^*^
Blood parameters		TSH	2.29 ± 1.97	2.18 ± 2.09	−0.345	.731
		FT3	4.31 ± 1.01	4.12 ± 0.88	−1.269	.206
		FT4	17.14 ± 3.25	16.52 ± 2.67	−1.322	.188
		K	3.61 ± 0.41	3.71 ± 0.38	1.557	.121
		Na	140.42 ± 4.65	140.88 ± 4.42	0.645	.520
		Fe	13.16 ± 7.79	12.31 ± 5.82	−0.785	.433
		Cl	104.17 ± 4.58	103.17 ± 5.18	−1.279	.203
		Ca	2.23 ± 0.15	2.24 ± 0.15	0.278	.781
		P	1.16 ± 0.24	1.16 ± 0.20	0.137	.891
		Mg	0.85 ± 0.11	0.87 ± 0.17	1.104	.271
		HCY	16.23 ± 9.71	14.25 ± 6.00	−1.520	.131
		CRP	10.39 ± 17.41	10.32 ± 20.58	−0.021	.983
		D‐dimer	0.77 ± 0.73	1.51 ± 3.14	2.105	.038^*^
		Uric acid	346.43 ± 140.55	344.20 ± 91.47	−0.116	.908
		HbA1c	6.12 ± 1.06	6.07 ± 1.00	−0.308	.758
		WBC	7.43 ± 2.71	7.18 ± 3.27	−0.519	.604
		RBC	4.24 ± 0.84	4.23 ± 0.70	−0.098	.922
		HGB	120.47 ± 15.76	126.14 ± 18.62	2.053	.042^*^
		PLT	229.77 ± 64.01	243.29 ± 130.75	0.805	.422
		TC	3.84 ± 1.26	4.08 ± 1.02	1.289	.199
		TG	1.44 ± 0.69	1.28 ± 0.61	−1.648	.101
		HDL	1.12 ± 0.28	1.17 ± 0.32	1.066	.288
		LDL	2.27 ± 0.88	2.27 ± 0.89	−0.016	.987
Baseline scale scores		PSQI	9.03 ± 4.01	8.23 ± 3.80	−1.272	.205
		MMSE	21.70 ± 4.99	23.10 ± 3.71	1.988	.049^*^
		HAMA	10.85 ± 3.97	8.14 ± 3.11	−4.847	< .001^*^
		HAMD	10.74 ± 5.04	8.08 ± 3.93	−3.660	< .001^*^
		NMSS	32.73 ± 12.63	28.37 ± 14.72	−1.983	.046^*^
		PDSS	84.80 ± 16.28	88.05 ± 27.34	−0.461	.647
		PAC‐SYM	12.65 ± 6.08	11.77 ± 7.97	0.398	.693
		Tinetti Balance	13.40 ± 1.39	14.86 ± 0.94	−4.026	< .001^*^
		Tinetti Gait	8.30 ± 0.73	11.32 ± 0.84	−12.36	< .001^*^
		BBS	41.50 ± 4.53	51.45 ± 4.47	−7.167	< .001^*^
Gait analysis from wearable devices	Timed up and go test	Mean step length (cm, $\bar{x}$ ± s)	37.11 ± 9.42	46.37 ± 10.10	−2.329	.029^*^
		Mean stride velocity (m/s, $\bar{x}$ ± s)	0.58 ± 0.17	0.83 ± 0.24	−3.003	.006^*^
		Mean Stride length (cm, $\bar{x}$ ± s)	73.25 ± 18.48	91.84 ± 20.80	−2.339	.028^*^
		Mean step frequency (step/min, $\bar{x}$ ± s)	94.67 ± 10.26	108.05 ± 13.53	−2.833	.009^*^
		Mean stride time (s, $\bar{x}$ ± s)	1.30 ± 0.14	1.14 ± 0.14	2.577	.017^*^
		Mean double support (s, $\bar{x}$ ± s)	0.26 ± 0.08	0.20 ± 0.04	2.052	.051
	Narrow lane test	Mean step length (cm, $\bar{x}$ ± s)	36.09 ± 9.19	40.88 ± 14.94	−1.015	.320
		Mean stride velocity (m/s, $\bar{x}$ ± s)	0.58 ± 0.17	0.74 ± 0.32	−1.421	.184
		Mean stride length (cm, $\bar{x}$ ± s)	71.82 ± 18.25	81.81 ± 29.80	−1.065	.298
		Mean step frequency (step/min, $\bar{x}$ ± s)	97.58 ± 10.44	108.29 ± 13.02	−2.283	.032^*^
		Mean stride time (s, $\bar{x}$ ± s)	1.26 ± 0.14	1.14 ± 0.13	2.164	.041^*^
		Mean double support (s, $\bar{x}$ ± s)	0.25 ± 0.07	0.23 ± 0.09	0.522	.606
	Turning test	Mean duration (s, $\bar{x}$ ± s)	30.96 ± 25.34	15.69 ± 7.47	−2.465	.014^*^
		Mean steps ($\bar{x}$ ± s)	38.95 ± 26.73	22.55 ± 9.29	−2.244	.025^*^
		Mean angular velocity (degree/s, $\bar{x}$ ± s)	39.02 ± 25.61	54.53 ± 21.02	−2.125	.040^*^
		Mean step duration (s, $\bar{x}$ ± s)	0.62 ± 0.20	0.64 ± 0.13	−0.319	.751

Abbreviations: PDSS, Parkinson's Disease Sleep Scale; CRP, C‐reactive protein; FT3, free triiodothyronine; FT4, free thyroxine; TSH, thyroid‐stimulating hormone; HbA1c, glycosylated hemoglobin; HCY, homocysteine; WBC, white blood cells; RBC, red blood cells; HGB, hemoglobin; PLT, platelets; BBS, Berg Balance Scale; MMSE, Mini‐Mental State Examination; HAMA, Hamilton Anxiety Scale; HAMD, Hamilton Depression Scale; PSQI, Pittsburgh Sleep Quality Index; PAC‐SYM, Patient Assessment of Constipation Symptoms; NMSS, Non‐motor Symptoms Scale; PDSS, Parkinson's Disease Sleep Scale.

* *p *< .05; *p*‐value is the result of *t*‐test and χ^2^ test.

**TABLE 3:** Comparison between the postural instability and gait disorder dominant (PIGD) and non‐PIGD groups in terms of Fazekas scale scores of white matter lesions.

Imaging Fazekas scales(MRI FLAIR)	Characteristic	PIGD	Non‐PIGD	*t*	*p*
A. Paraventricular white matter (PVWM)	0	0	3	11.707	.005^*^
	1	10	29		
	2	27	24		
	3	36	30		
B. Deep white matter (DWM)	0	26	43	9.650	.020^*^
	1	32	20		
	2	16	17		
	3	1	6		
Fazekas	1	23	43	8.888	.012^*^
	2	37	24		
	3	13	19		

*p < .05; *p*‐value is the result of t‐test and χ2 test.

**FIGURE 1:**
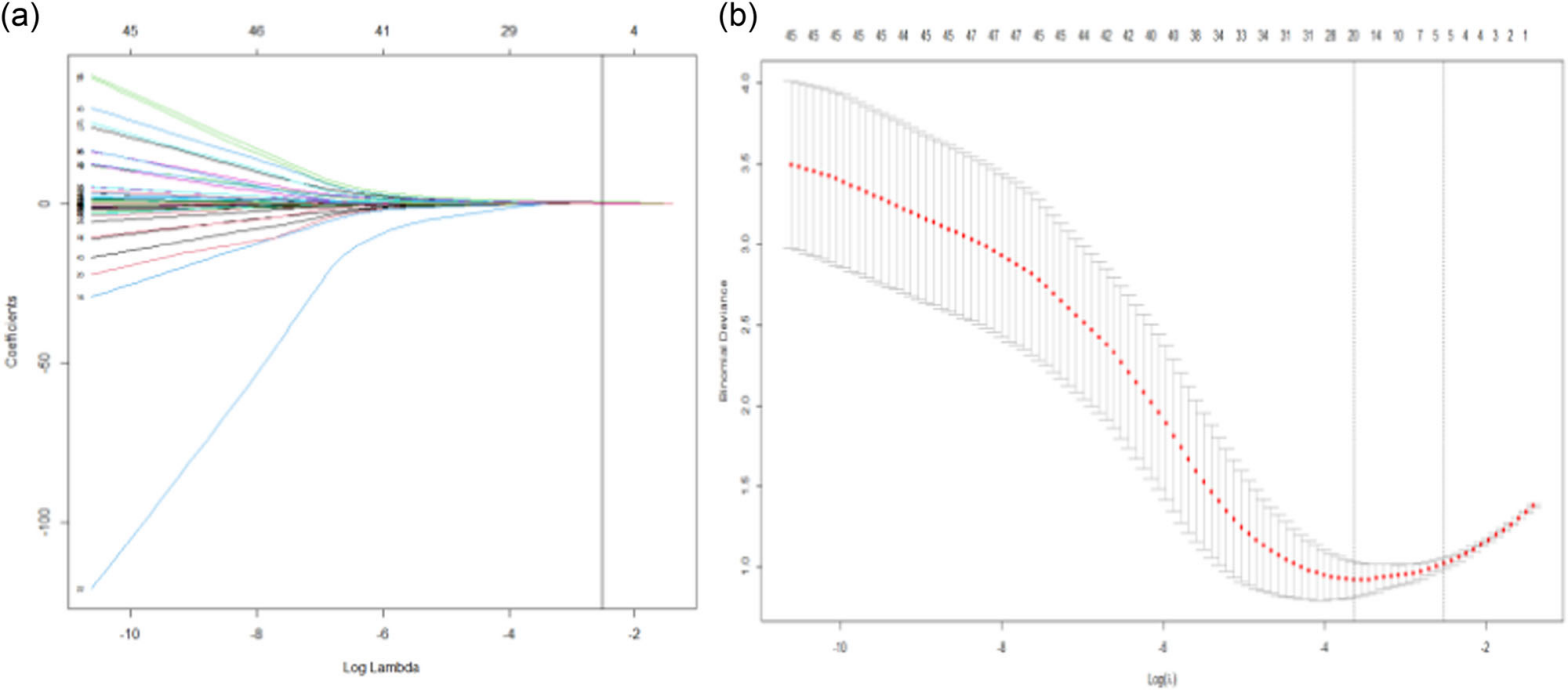
Coefficient curves (a) and variables after least absolute shrinkage and selection operator (LASSO) regression (b) (the vertical bar indicates log [lambda = 1 SE]).

**FIGURE 2:**
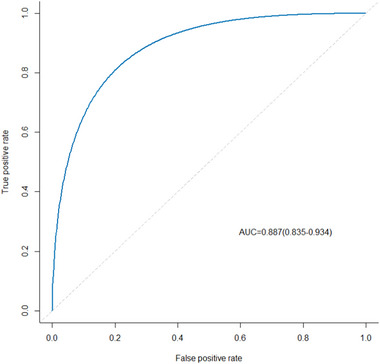
Receiver operating characteristic (ROC) curve for the postural instability and gait disorder dominant (PIGD) prediction model.

The univariate logistic regression analysis showed that the HGB level, MMSE score, HAMA score, HAMD score, PVWM, H‐Y stage, and non‐tremor mode of onset were significantly associated with the occurrence of PIGD (all *p* < .05) (Table 4). To reduce multicollinearity, LASSO regression was used for screening variables (Figure 1). Then multivariate logistic regression analysis showed that the MMSE score (β = −0.172; 95% CI, 0.75 to 0.97, *p *= .006), HAMA score (β = 0.234; 95% CI, 1.07 to 1.41, *p < *.001*)*, H‐Y stage (β = 0.820; 95% CI, 1.48 to 4.48, *p* < .001), PVWM score (β = 0.824; 95% CI, 1.31 to 3.95, *p* = .003), and non‐tremor mode of onset (β = 1.134; 95% CI, 4.34 to 5.16, *p* < .001) were significantly associated with the occurrence of PIGD (all *p* < .05) (Table 5). Cognitive dysfunction, anxiety status, H‐Y stage, PVWM score, and non‐tremor type mode of onset were found to be independent risk factors for PIGD. The regression equation for predicting PIGD was as follows: Y = −3.128 − 0.172 × (MMSE) + 0.234 × (HAMA) + 0.820 × (H‐Y) + 1.134 × (mode of onset) + 0.824 × (PVWM) (Table 5). The ROC curve for this model is shown in Figure 2, and the AUC value was 0.887 (95% confidence interval [CI]: 0.835−0.934).

**TABLE 4:** Univariate logistic regression analysis for postural instability and gait disorder dominant (PIGD).

Characteristic		*Β*	S.E.	*z*	OR (95% CI)	*p*
Baseline clinical characteristics	Sex	0.073	0.323	0.225	1.08 (0.57, 2.03)	.822
	Age	−0.009	0.016	−0.578	0.99 (0.96, 1.02)	.563
	Occupation	0.205	0.165	1.239	1.23 (0.89, 1.71)	.215
	Education	−0.119	0.147	−0.814	0.89 (0.66, 1.18)	.416
	Marital status	0.514	0.382	1.347	1.67 (0.8, 3.6)	.178
	Height	0.019	0.022	0.864	1.02 (0.98, 1.06)	.388
	Weight	0.015	0.018	0.842	1.02 (0.98, 1.05)	.400
	BMI	0.005	0.039	0.133	1.01 (0.93, 1.09)	.894
	H‐Y	0.891	0.176	5.078	2.44 (1.75, 3.49)	< .001^*^
	Course	−0.035	0.042	−0.815	0.97 (0.88, 1.05)	.415
	Diabetes	0.286	0.369	0.773	1.33 (0.64, 2.76)	.439
	CHD	0.381	0.43	0.885	1.46 (0.63, 3.45)	.376
	HBP	0.204	0.326	0.626	1.23 (0.65, 2.34)	.531
	HLP	−0.243	0.367	−0.663	0.78 (0.38, 1.6)	.507
	Smoking status	0.025	0.418	0.06	1.03 (0.45, 2.33)	.952
	Alcohol consumption	0.629	0.553	1.137	1.88 (0.64, 5.85)	.256
	MDS‐UPDRSI	−0.015	0.072	−0.216	0.98 (0.85, 1.13)	.829
	MDS‐UPDRSII	−0.018	0.021	−0.851	0.98 (0.94, 1.02)	.395
	MDS‐UPDRSIII	−0.002	0.015	−0.138	1 (0.97, 1.03)	.890
	Mode of onset Tremor	1.13	0.502	2.245	3.09 (1.15, 8.35)	.025^*^
	Non‐tremor	2.41	0.411	5.849	11.1 (5.09, 25.67)	< .001^*^
Blood parameters	TSH	0.027	0.078	0.346	1.03 (0.88, 1.2)	.729
	FT3	0.216	0.171	1.262	1.24 (0.89, 1.75)	.207
	FT4	0.072	0.055	1.315	1.07 (0.97, 1.2)	.189
	TC	−0.183	0.142	−1.285	0.83 (0.63, 1.1)	.199
	TG	0.408	0.25	1.631	1.5 (0.93, 2.48)	.103
	HDL	−0.569	0.535	−1.063	0.57 (0.19, 1.59)	.288
	LDL	0.003	0.181	0.016	1 (0.7, 1.43)	.987
	K	−0.638	0.414	−1.539	0.53 (0.23, 1.18)	.124
	Na	−0.023	0.035	−0.647	0.98 (0.91, 1.05)	.518
	Fe	0.019	0.024	0.783	1.02 (0.97, 1.07)	.434
	Cl	0.042	0.033	1.271	1.04 (0.98, 1.12)	.204
	Ca	−0.297	1.061	−0.28	0.74 (0.09, 5.99)	.780
	P	−0.101	0.73	−0.138	0.9 (0.21, 3.81)	.891
	Mg	−1.325	1.229	−1.078	0.27 (0.02, 2.52)	.281
	HCY	0.033	0.021	1.53	1.03 (0.99, 1.08)	.126
	CRP	0	0.008	0.021	1 (0.98, 1.02)	.983
	D‐dimer	−0.298	0.172	−1.73	0.74 (0.51, 0.96)	.084
	UA	0	0.001	0.121	1 (1, 1)	.904
	WBC	0.028	0.053	0.521	1.03 (0.93, 1.14)	.602
	RBC	0.02	0.208	0.098	1.02 (0.68, 1.54)	.922
	HGB	−0.019	0.01	−2.008	0.98 (0.96, 1)	.045^*^
	PLT	−0.001	0.002	−0.773	1 (0.99, 1)	.440
	HbA1c	0.048	0.155	0.311	1.05 (0.77, 1.43)	.756
Baseline scale scores	PSQI	0.052	0.041	1.268	1.05 (0.97, 1.14)	.205
	MMSE	−0.194	0.049	−3.996	0.82 (0.74, 0.9)	< .001^*^
	HAMA	0.224	0.053	4.238	1.25 (1.13, 1.4)	< .001^*^
	HAMD	0.132	0.038	3.463	1.14 (1.06, 1.23)	< .001^*^
	NMSS	0.023	0.012	1.95	1.02 (1, 1.05)	.051

Abbreviations: MDS‐UPDRS, Movement Disorder Society Unified‐Parkinson Disease Rating Scale; UA, uric acid; TSH, thyroid‐stimulating hormone; FT3, free triiodothyronine; FT4, free thyroxine; HCY, homocysteine; CRP, C‐reactive protein; WBC, white blood cells; RBC, red blood cells; HGB, hemoglobin; PLT, platelets; HbA1c, glycosylated hemoglobin; MMSE, Mini‐Mental State Examination; HAMA, Hamilton Anxiety Scale; HAMD, Hamilton Depression Scale; PSQI, Pittsburgh Sleep Quality Index; NMSS, Non‐motor Symptoms Scale.

* *p *< .05; *p*‐value is the result of univariate logistic regression.

**TABLE 5:** Multivariate logistic regression analysis for postural instability and gait disorder dominant (PIGD).

Characteristic	*Β*	S.E.	Wald χ2	OR (95% CI)	*p*
MMSE	−0.172	0.063	−2.416	0.86 (0.75,0.97)	.006^*^
HAMA	0.234	0.072	2.774	1.22 (1.07,1.41)	< .001^*^
H‐Y	0.820	0.216	3.720	2.23 (1.48,3.48)	< .001^*^
Mode of onset(Non‐Tremor)	1.134	0.523	4.680	11.56 (4.34,5.16)	< .001^*^
PVWM	0.824	0.281	8.601	2.28(1.31,3.95)	.003^*^
Constant	−3.128	1.644	−1.903	0.04 (0.00,1.03)	.057

* *p *< .05; *p*‐value is the result of multivariate logistic regression.

## 4 DISCUSSION

This study showed that PD patients had lower Tinetti Balance, Tinetti Gait, and BBS scores than the control group, suggesting they had a weaker ability to balance. We used a GYENNO MATRIX wearable sensor system for three trials and found that multiple spatiotemporal gait parameters were changed in PD patients. The results also show that subcortical structures were altered in PD patients, which affected the gait automation process. This, in turn, can cause decreased attention and impaired sensory‐motor integration processes in PD patients, resulting in a decreased compensatory ability to control gait through active movement and, thus, increased gait variability (Bohnen & Jahn, 2013). This can cause gait changes in PD patients, such as a reduced swing amplitude and pace of both hands and slow pace. In this study, the PIGD group presented with more severe gait problems in different wearable sensor trials than those in the non‐PIGD group. Patients with PIGD had lower scores on the Tinetti Balance Scale, Gait Scale, and BBS, as well as a worse ability to balance. These results indicate that PD patients with PIGD may have more severe damage to the subcortical motor structure and more prominent disturbances in the sensorimotor integration process than those without PIGD, resulting in worse performances in stride speed, stride cycle, stride length, the single support phase, the double support phase, and step width, as well as in other fine aspects of gait. Previous studies (Cao et al., 2020; Lamont et al., 2018; Rochester et al., 2014) applied sensors for gait testing in PD patients and showed that the increase in gait variability reflects an increase in gait instability, which can be detected early in PD and is considered to be a marker of disease progression. As gait disorder worsens, the duration of the dual support phase increases and motor automation is further compromised, leading to the fragmentation of motor function, including the impairment of turning and starting (Son et al., 2017). Our results are similar to those of previous studies. The wearable sensor system used in this study has been proven to be a valuable diagnostic tool for a gait analysis system that can objectively assess gait parameters in PD (Lin et al., 2023). Our study quantified the fine gait features of PD patients using a wearable device and also proved that wearable gait analyzers have several advantages and practical uses in gait evaluation.

In this study, we used wearable devices to evaluate the gait of patients, and we found that the average H‐Y stage of PIGD patients was significantly higher than that of non‐PIGD patients. A previous study (Yang et al., 2016) showed that the H‐Y stage is associated with gait, and another study Nohelova et al. (2021) showed that the H‐Y stage is an important predictor of gait in PD under auditory dual‐task conditions. Vila et al. (Vila et al., 2021) used a wearable motion sensor to find that H‐Y stages III and IV were associated with a significantly worse gait compared to stages I and II. At present, H‐Y staging alone cannot be used to determine the evolution of spatiotemporal and kinematic parameters and their impact on the deterioration of walking patterns. Therefore, devices such as gait analyzers, portable sensors, and wearable devices have been developed to provide new methods for the early identification of PD gait disorders. Our results are consistent with those of previous studies. We confirmed that the presence of gait disorder was positively correlated with H‐Y stage.

The study showed that among PD patients with different onset modes, patients with the non‐tremor onset mode were more likely to develop gait disorders than those with the tremor onset mode. The proportion of patients with non‐tremor onset was highest in the PIGD group. Clinically, the onset mode of PD patients can be divided into tremor‐dominant and non‐tremor types (Jankovic et al., 1990). Álvarez et al. (Álvarez et al., 2020) showed that compared with PD patients with tremors, patients with non‐tremor onset had slightly higher developmental balance impairment and increased gait disorder as the disease progressed. Thenganatt et al. (Thenganatt & Jankovic, 2014) demonstrated that the occurrence of non‐tremor onset in PD patients with frozen gait resulted in these patients having a slower pace and taking shorter steps. A possible reason for this is that the loss of neurons in the substantia nigra is less severe and the progression of neuron degeneration is slower in PD patients with tremor‐type onset. Therefore, the progression of PD with tremor type into gait disorder is slower. Our results are consistent with those of previous studies, but there remains a lack of in‐depth research on the pathogenesis of gait disorders.

This study showed that the NMSS scores of patients in the PIGD group were significantly higher than those in the non‐PIGD group. This scale contains 30 items, including measures of non‐motor symptoms such as sleep, mood, cognition, and gastrointestinal function. A related study (Wu et al., 2022) compared NMSS scores in two groups of patients with different subtypes of PD and found that patients in the PIGD group had higher NMSS scores than those in the non‐PIGD group. Another study showed (Zhang et al., 2023) that PD patients with higher NMSS scores have more severe gait impairment. Our results are consistent with those of previous studies. Moreover, patients with PIGD often have a higher striatal dopamine consumption, which can lead to higher muscle stiffness (Rossi et al., 2010).

This study showed that compared with the control group, the HAMA and HAMD scores were higher in PD patients, and our further study showed that the HAMA and HAMD scores of patients in the PIGD group were significantly higher than those in the non‐PIGD group. PD patients often have anxiety and depression, with an average incidence of 20%−50%, which is higher than that in the general population (Ray & Agarwal, 2020). Many studies have suggested that gait characteristics vary according to one's emotional state. For example, decreased walking speed and arm swing may be associated with depression (Michalak et al., 2009), and anxiety can aggravate motor symptoms and may lead to aggravated cognitive symptoms (Macht et al., 2005). A previous study (Taximaimaiti & Wang, 2021) demonstrated that anxiety and depression are risk factors of frozen gait. Kincses et al. (Kincses et al., 2017) found that, in the multitask walking test, a higher depression score was associated with a slower walking speed and shorter stride amplitude in PD patients. Ehgoetz et al. (Ehgoetz et al., 2014) used virtual reality technology to induce anxiety in PD patients and assessed its effect on gait in PD, thus demonstrating that anxiety increases the occurrence of gait disorders. Avanzino et al. (Avanzino et al., 2018) demonstrated that PIGD in PD patients can vary according to one's mood, with depressive states having the largest impact on persistent gait disorders and anxiety states primarily affecting paroxysmal gait disorders. Our conclusions are consistent with those of previous studies and confirm the high incidence of anxiety and depression, as well as its impact on gait disorders in PD patients.

In this study, the PAC‐SYM score of PD patients was significantly higher than that of the healthy control group. There was no difference in the PAC‐SYM score between the PIGD and non‐PIGD groups. Constipation is one of the most common and disabling non‐motor symptoms in PD patients (Barone et al., 2009). A previous study showed that (Chen et al., 2015) the prevalence of constipation in PD patients is 50%, while that in patients with constipation before diagnosis is 20%. It has been demonstrated (Adams‐Carr et al., 2016) that constipation is one of the risk factors for PD. People with constipation had a 2.27‐fold increased risk of PD compared to those without constipation. Our results are generally consistent with those of previous studies. However, our results further indicate that constipation is not directly related to the occurrence of Parkinson's gait disorder, so whether constipation is related to gait disorder still needs further investigation.

This study also showed that compared with the control group, patients in the PD group had lower MMSE scores and more serious cognitive dysfunction. Furthermore, our research showed that patients in the PIGD group had significantly more serious cognitive dysfunction than those in the non‐PIGD group. Cognitive dysfunction is common in PD patients (Nicoletti et al., 2019). Monaghan et al. (Monaghan et al., 2023) demonstrated that PD patients with freezing of gait had worse memory, execution, and attention in a range of cognitive domains compared to PD patients without freezing of gait. Moreover, previous studies have shown (Konno et al., 2018; Reijnders et al., 2009) that PD patients with PIGD also have faster early cognitive decline and progression of dementia. Baiano et al. (Baiano et al., 2020) demonstrated that the PIGD type occurs more frequently in PD patients with dementia compared to the other types. These results suggest that there is a potential bidirectional relationship between gait disorders and cognitive dysfunction in PD patients, which is mutually causal. Alison et al. (Yarnall et al., 2014) stated that the cortical areas involved in motor, cognitive, limbic, and associative functions have lower gray matter volumes in PIGD than in non‐PIGD. Therefore, we believe that the development of gait disorder in PD patients is related to the abnormality of the basal ganglia‐thalamic‐cortical ring in cognitive function. Our results are generally consistent with those of previous studies. The reasons for the association between the development of gait disorder and cognitive decline in patients are not fully understood.

This study showed that patients in the PIGD group had significantly lower hemoglobin levels than those in the non‐PIGD group. Tsu‐Kung et al. (Lin et al., 2020) compared PD patients with normal nutrition and malnourishment and found that the hemoglobin level was significantly lower in the malnourished risk group than in the nutritionally normal group. However, there was no statistically significant difference between the two groups of patients in the score of the third part of the MDS‐UPDRS. Ongun (Ongun, 2018), in contrast, has demonstrated that the motor and non‐motor function, disease duration, and PD severity are associated with nutritional status. The quality of life in PD is also influenced by changes in nutritional status. It is hypothesized that malnutrition may lead to decreased muscle strength, muscle mass, and spasms. In turn, spasms may lead to increased muscle tone and decreased balance. Our conclusions are both similar to and different from previous studies.

In this study, the D‐dimer level in PIGD patients was significantly lower than that in the non‐PIGD group. Previous research (Ospina‐Romero et al., 2018) has shown that the thrombomodulin activity is related to HCY, and a high HCY can inhibit thrombomodulin, resulting in the blockage of the activation pathway of anticoagulant protein C, which is manifested by the increased blood D‐dimer level. A previous study also showed that the formation of HCY is involved in the methylation process of levodopa (Paul & Borah, 2016). High HCY in PD is induced by levodopa intake, which can partially explain the increased D‐dimer level in PD patients. At present, the relationship between D‐dimer level and gait disorder in PD has not been explored, so further prospective studies are needed in the future.

This study showed that, compared with the non‐PIGD group, patients in the PIGD group had higher scores in PVWM, DWM scores, and Fazekas grading, revealing that patients in the PIGD group had more serious lesions of white matter. Acharya et al. (Acharya et al., 2007) showed that white matter lesions mainly affect the axial motor function of PD patients. Gallardo et al. (Gallardo et al., 2014) showed that PD patients with frozen gait had higher Fazekas scores. Furthermore, a relevant study (Herman et al., 2013) has shown that in patients with different subtypes of PD, with either tremor or non‐tremor onset, the degree of white matter lesions was not correlated with the clinical feature score of the patient's gait. Moreover, Song et al. (Song et al., 2013) showed, in a follow‐up study of PD patients, that ischemic white matter changes accompanied by mild and asymptomatic changes had no significant impact on the progression of exercise severity in PD patients. Another study (Veselý et al., 2016) has shown that white matter lesions, especially periventricular lesions, are associated with more severe Parkinson's symptoms. Kelly et al. (Kelly et al., 2015) found that periventricular lesions were more likely to impair cognitive function, thereby affecting more severe gait disorders. However, it has also been suggested (Balash & Korczyn, 2007) that vascular lesions in deep white matter may affect the striatum and lead to gait disorders. Our conclusions are both similar to and different from those of previous studies. It is well known that white matter lesions are an important factor affecting gait and cognition in the elderly. However, at present, the relationship between white matter lesions in different parts of the brain and gait disorders in PD remains controversial, so further studies are needed.

We analyzed the factors affecting PD gait disorder and found that cognitive dysfunction, anxiety, H‐Y stage, PVWM score, and mode of onset (non‐tremor type) were independent risk factors for PIGD. The more severe the cognitive dysfunction (i.e., the lower the MMSE score) is, the more severe the anxiety (i.e., the higher the HAMA score) is, the higher the H‐Y grade is, the non‐tremor onset of PD is, the more severe the periventricular white matter lesions (the higher the PVWM score) are, the more likely PIGD is to occur. Our findings are largely consistent with those of previous studies. Whether the imaging PVWM score is related to PD gait disorder remains controversial. Herein, we established a statistical regression model for the prediction of PIGD‐related factors and evaluated the model through ROC curves and AUC values. The predictive effect of the model was confirmed to be good. The prediction of the risk of PD gait disorders is essential for reducing falls and other accidents. In the future, we will perform clinical validation to assess the accuracy of the model, as well as establish the optimal model.

## 5 CONCLUSION

PD patients have a higher incidence of gait disorders than the healthy population. Patients with PD combined with PIGD experience more severe gait problems and more severe impairment of gait automation in different test tasks than those without PIGD. Cognitive dysfunction, anxiety status, PVWM score, H‐Y stage, and non‐tremor mode of onset were independent risk factors for PIGD. In the future, it is expected that gait disorders in PD can be quantitatively assessed based on wearable devices, and prediction models can be used to effectively identify PIGD in the PD population and develop targeted interventions to reduce the risk of PIGD. Such evidence may also be valuable for understanding the potential mechanisms underlying gait disorders in PD.

## AUTHOR CONTRIBUTIONS

**Hongyin Tang**: Writing—original draft; conceptualization. **Xianglian Liao**: Software. **Jian Yao**: Data curation. **Yilan Xing**: Investigation. **Xin Zhao**: Investigation. **Weibin Cheng**: Investigation. **Tianxiang Gu**: Software. **Yan Huang**: Data curation. **Guang Xu**: Validation. **Ping Luan**: Formal analysis; supervision; resources. **Junzhang Tian**: Methodology. **Guihua Li**: Writing—review and editing; writing—original draft; project administration; visualization.

### PEER REVIEW

The peer review history for this article is available at https://publons.com/publon/10.1002/brb3.3440

## Data Availability

The data that support the findings of this study are openly available in Pubmed at https://pubmed.ncbi.nlm.nih.gov/.
